# Helping the self help others: self-affirmation increases self-compassion and pro-social behaviors

**DOI:** 10.3389/fpsyg.2014.00421

**Published:** 2014-05-12

**Authors:** Emily K. Lindsay, J. David Creswell

**Affiliations:** Department of Psychology, Carnegie Mellon UniversityPittsburgh, PA, USA

**Keywords:** self-affirmation, self-compassion, pro-social behavior, social values, self-concept, writing

## Abstract

Reflecting on an important personal value in a self-affirmation activity has been shown to improve psychological functioning in a broad range of studies, but the underlying mechanisms for these self-affirmation effects are unknown. Here we provide an initial test of a novel self-compassion account of self-affirmation in two experimental studies. Study 1 shows that an experimental manipulation of self-affirmation (3-min of writing about an important personal value vs. writing about an unimportant value) increases feelings of self-compassion, and these feelings in turn mobilize more pro-social behaviors to a laboratory shelf-collapse incident. Study 2 tests and extends these effects by evaluating whether self-affirmation increases feelings of compassion toward the self (consistent with the self-compassion account) or increases feelings of compassion toward others (an alternative other-directed compassion account), using a validated storytelling behavioral task. Consistent with a self-compassion account, Study 2 demonstrates the predicted self-affirmation by video condition interaction, indicating that self-affirmation participants had greater feelings of self-compassion in response to watching their own storytelling performance (self-compassion) compared to watching a peer’s storytelling performance (other-directed compassion). Further, pre-existing levels of trait self-compassion moderated this effect, such that self-affirmation increased self-compassionate responses the most in participants low in trait self-compassion. This work suggests that self-compassion may be a promising mechanism for self-affirmation effects, and that self-compassionate feelings can mobilize pro-social behaviors.

## INTRODUCTION

Reflecting on an important personal value in a self-affirmation exercise has been shown to have a broad range of beneficial effects across over 225 published studies (for reviews, see Sherman and Cohen, 2006; Cohen and Sherman, 2014). For example, a brief self-affirmation of an important personal value, such as writing about why you value friends and family, has been shown to buffer many different threats to the self, such as reducing rumination in response to failure feedback (Koole et al., 1999), lowering stress reactivity to social evaluation (Creswell et al., 2005, 2013), and in mitigating the effects of stereotype threat on academic performance in classroom settings (Cohen et al., 2006; Miyake et al., 2010). Despite this large body of work, the mechanisms of self-affirmation are not well specified, and currently two theoretical perspectives have been offered to explain how self-affirmation exerts its effects. A longstanding theoretical perspective posits that self-affirmation boosts one’s self-image for coping with self-threats (Sherman and Cohen, 2006). Although some studies provide support for this *self-resources* account (e.g., increasing self-esteem and self-regulatory strength; Schmeichel and Vohs, 2009; Sherman and Hartson, 2011), empirical support for this mechanistic explanation has been limited (Sherman and Cohen, 2006; Crocker et al., 2008). In contrast, a more recent theoretical perspective offers that self-affirmation enables one to *transcend* self-image concerns by increasing other-directed feelings (Crocker et al., 2008). In one influential study, Crocker et al. (2008) showed that affirmed participants reported greater feelings of love and connection, and that these feelings statistically explained how self-affirmation reduced defensiveness to a threatening health message.

Here we test a novel self-compassion account that links these two theoretical self-affirmation perspectives. Specifically, we posit that self-affirmation activities increase feelings of self-compassion, characterized by increased self-directed feelings of sympathy and love, and reductions in feelings of vulnerability and criticism (cf. Neff, 2003a; Leary et al., 2007). Our self-compassion account is consistent with the existing theoretical frameworks for self-affirmation: increasing self-compassion is one form of boosting one’s self-image (i.e., the self-resources perspective), and is associated with increased feelings of love and connection (i.e., the self-transcendence perspective; cf. Neff, 2003a). But this self-compassion perspective provides new specificity to these previous theoretical accounts by positing that the self-affirmation self-image boost is about feeling more compassion toward the self (and is not a general self-esteem boost as suggested by the self-resources perspective; Neff and Vonk, 2009), and that compassionate feelings engendered by self-affirmation are not other-directed (as suggested by the self-transcendence perspective), but directed toward the self. It is difficult, however, to disentangle whether these feelings stimulated through values affirmation are directed toward the self or toward others, and furthermore, it’s possible that compassionate feelings toward the self may generate compassion for others. Indeed, one important aspect of a self-compassionate attitude is the recognition of oneself as part of the human condition (Neff, 2003a); this sense of shared humanity may be encouraged by writing about important values, consistent with the self-transcendence perspective, but we suggest that the source of these feelings is a boost in self-compassion.

The current work was inspired by the work of Crocker et al. (2008) suggesting that self-affirmation may increase feelings of love and social connection. Building on previous studies suggesting that feelings of love and compassion may foster helping behavior (Mikulincer et al., 2005; Piff et al., 2010), Study 1 tests the prediction that self-affirmation will increase feelings of self-compassion, which in turn will increase pro-social behavior. Although no previous studies have tested self-compassion as a mechanism, one recent developmental psychology study suggests that self-affirmation can increase pro-social feelings and teacher-rated behaviors among adolescent students, particularly among students who displayed higher levels of antisocial behavior (Thomaes et al., 2012). Another set of studies showed that self-affirmation increased pro-social behavior only when paired with feelings of moral elevation (Schnall and Roper, 2012). These studies suggest that self-affirmation may impact pro-social behavior through multiple and possibly yet unidentified processes. In Study 2, we test the specificity of the self-compassion account by testing whether self-affirmation increases feelings of compassion toward the self (self-compassion) as opposed to fostering feelings of compassion toward a stranger (other-directed compassion), using a validated behavioral task of self-compassion (Leary et al., 2007, Study 4).

We hypothesized that self-affirmation would increase feelings related to self-compassion, and that these feelings would mediate the effects of self-affirmation on increased pro-social behavior to a laboratory shelf-collapse incident (Study 1). In Study 2, we tested the specificity of self-affirmation on compassion, predicting that self-affirmation would increase feelings of self-compassion but not other-directed compassion in evaluating a mildly embarrassing video (their own “self” video vs. a peer “other” video). As previous studies indicate that self-affirmation may be particularly effective at buffering threats to participants who are the most resource deficient (e.g., among ego-depleted participants: Schmeichel and Vohs, 2009; participants with high levels of anti-social behavior: Thomaes et al., 2012), we hypothesized a moderating role of trait self-compassion in Study 2, such that self-affirmation would be more likely to increase self-compassionate feelings (to watching the “self” video) among participants who had pre-existing low levels of trait self-compassion.

## STUDY 1 METHOD

### PARTICIPANTS

Fifty-eight Carnegie Mellon students (*N* = 58) were recruited (67% female; age: *M* = 19.71 years, SD = 2.2; 52% Caucasian, 29% Asian, 8% African American, 6% Mixed, 2% Latino, 4% Other) in exchange for course credit or $8. The statistical software package G^*^Power indicated that a total sample size of 52 participants would provide 80% power to detect large main effects of self-affirmation (consistent with previous research indicating large effects of self-affirmation: McQueen and Klein, 2006; Crocker et al., 2008). This research was approved by the Carnegie Mellon University Institutional Review Board, and all volunteers provided written informed consent. Six participants were dropped prior to analysis: three did not follow study instructions, and three due to technical problems.

### PROCEDURE AND DESIGN

Participants were told that the purpose of the study was to investigate the effects of mood on language use. Participants completed a two-part self-affirmation manipulation and a pre- and post-affirmation state affect checklist. Following standard procedures for manipulating self-affirmation (Cohen et al., 2006; McQueen and Klein, 2006), participants rank-ordered a list of 11 values (e.g., artistic skills, independence) in terms of their personal importance. Participants were then randomly assigned to write for 3 min about their top-ranked value and why it was personally meaningful (self-affirmation condition) or why their bottom-ranked value might be important to someone else (control condition). Affirmation and control writing sheets were pre-randomized and administered so that only subject number and instructions were visible to experimenters, thus blinding them to study condition.

**FIGURE 1:**
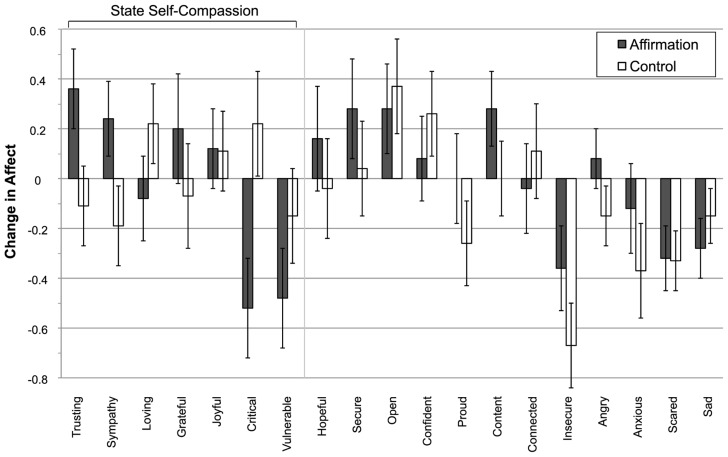
**Changes in individual state affect items in the affirmation and control groups in Study 1.** The y-axis describes the pre–post affirmation change score value for each group. Values above 0 on the y-axis refer to an increase in pre–post affirmation affect. Error bars refer to ±1 SE of the mean.

Participants rated affect items “right now” before and after the affirmation exercise on a 5-point Likert scale (*not at all* to *extremely*; Watson et al., 1988). Affect items were selected based on Crocker et al. (2008; **Figure 1**), and allowed us to test for changes in feelings related to the construct of self-compassion (e.g., greater sympathy, less criticism; cf. Neff, 2003a) and to test single item measures of social connection previously implicated in self-affirmation effects (e.g., love; Crocker et al., 2008; see Measures). To ensure participants did not link the affirmation activity with the subsequent pro-social dependent measure and to reduce suspicion, participants then completed a 12-item bogus sentence-unscrambling “language” task (consistent with our cover story).

To test the hypothesis that self-affirmation increases pro-social behavior, participants provided two measures of helping behavior (see Measures). First, participants completed an indirect survey measure of hypothetical charitable giving. Second, participants’ helping behavior was measured in response to a surprise shelf-collapse incident that occurred while they completed some final questionnaires. The experimenter waited 1 min after the completion of these questionnaires before re-entering the room to pick up the fallen shelf items if participants had not already done so. Participants were then probed for suspicion (none were suspicious about the shelf-collapse incident), debriefed, and dismissed.

### MEASURES

#### Affect scales

In addition to testing for changes from pre- to post-affirmation in the individual affect items *loving* and *connected* (Crocker et al., 2008), we formed a composite measure indexing self-compassion from participants’ individual state affect ratings. The *Feelings of State Self-Compassion* measure reflecting theoretical accounts of compassion was administered before (α = 0.62) and after (α = 0.75) affirmation writing. The items on this *Feelings of State Self-Compassion* measure included *critical* (reverse-scored), *sympathy*, *grateful*, *trusting*, *vulnerable* (reverse-scored), *joyful* and *loving*. This pre- and post-assessment allowed us to test for condition differences in *change* in state self-compassion; we calculated a post-pre change score in feelings of state self-compassion.

#### Reported charitable giving

As an indirect measure of pro-social behavior, participants completed a spending survey, allocating 100% of one’s income to nine categories (bills, food, clothing, luxury items, recreation, charitable giving, travel, gifts, housing). Importantly, the category of charitable giving was used as a covert measure of pro-social behavior (Piff et al., 2010, Study 2), with higher percentages indicating greater desire for charitable spending.

#### Helping behavior shelf-collapse incident

To directly measure helping behavior, we designed a surprise shelf-collapse incident in the lab. Specifically, the experimenter instructed the participant to complete some questionnaires (another affect scale and the demographics measure) while she set up for another participant in an adjacent room. A non-bracketed shelf containing paper clips, pens, and alcohol swabs hung on the door to the experimental room (about 3 m from the seated participant), such that when the experimenter exited the room and closed the door, this shelf (and its contents) crashed to the ground. The experimenter (blind to subject condition) observed participants’ reactions using an unobtrusive video camera, and scored participants’ helping behavior on a 9-point Likert Scale (scale anchors: 0 = provided no help at any time, 4 = participant informs experimenter of incident upon experimenter’s return and then helps experimenter pick up items, 8 = immediate helping with fallen items), with higher scores indicating more helping behavior. All participants noticed the shelf-collapse.

## STUDY 1 RESULTS

Self-affirmation (vs. control) participants had a significantly greater increase in feelings of state compassion pre–post writing. Specifically, a one-way ANOVA (condition: self-affirmation or control) revealed a significant difference in state compassionate feelings [*F*(1,50) = 4.23, *p* = 0.05, η^2^ = 0.08]: self-affirmation participants had a greater pre-post-writing change in state compassion (*M* = 1.84, SD = 3.3) compared to control participants (*M* = -0.11, SD = 3.52). A 2 (condition: self-affirmation or control) × 2 (time: pre- and post-writing) repeated measures ANOVA [affirmation X time interaction *F*(1,50) = 4.23, *p* = 0.05, η^2^ = 0.08] yielded the same effect as the one-way ANOVA using the affect change score: self-affirmation increased state compassion pre-post-writing, compared to the control group.

It was predicted that self-affirmation increases pro-social behavior. This hypothesis was tested in two ways. First, it was predicted that affirmed participants would indicate a desire to give more of their income to charities on the spending survey. A significant positive relationship between family income and charitable giving was observed in this sample (*r* = 0.31, *p* = 0.02), so family income was used as a covariate in this analysis. A one-way (condition: self-affirmation, control) ANCOVA yielded a significant main effect on percentage of income allocated to charitable donations [*F*(1,50) = 5.90, *p* = 0.02, η^2^ = 0.11]. Specifically, affirmation participants indicated a greater desire for charitable giving (*M* = 6.58%, SD = 3.66) compared to control participants (*M* = 4.24%, SD = 3.41). Without controlling for family income, the effect of self-affirmation on charitable giving did not reach statistical significance [*F*(1,50) = 2.21, *p* = 0.14, η^2^ = 0.04]. Second, it was predicted that self-affirmed participants would exhibit greater helping behavior to the shelf-collapse incident. Indeed, a one-way ANOVA confirmed that self-affirmation participants helped more (*M* = 3.92, SD = 3.02) than control participants in response to the shelf-collapse incident (*M* = 2.33, SD = 2.2) [*F*(1,46) = 4.32, *p* = 0.04, η^2^ = 0.09].

### STATE COMPASSION IS A MECHANISM FOR SELF-AFFIRMATION EFFECTS

**FIGURE 2:**
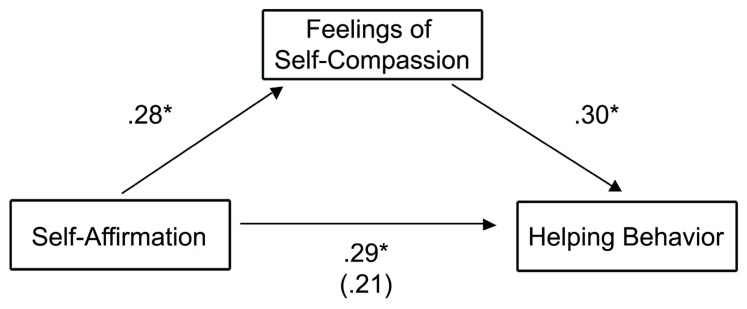
**Change in state self-compassion mediates the effect of the self-affirmation manipulation on helping behavior to a shelf-collapse incident in Study 1.** To determine if compassion predicted greater helping behavior, the proposed mediating variable (the measure of composite self-compassion) and the predictor variable (the self-affirmation condition) were entered simultaneously in a multiple regression equation predicting the outcome variable (helping behavior score). Numbers represent beta coefficients, with parentheses representing beta coefficients when feelings of self-compassion and the self-affirmation treatment variable are entered simultaneously in a multiple regression analysis. *p < 0.05.

Mediation analyses (Baron and Kenny, 1986) were consistent with the prediction that increases in feelings of compassion explain how self-affirmation increases helping behavior to the shelf-collapse incident. A series of multiple regression analyses showed that change in state self-compassion was an intervening variable for the effects of self-affirmation on increasing pro-social behavior to the shelf-collapse incident. As predicted, greater feelings of compassion predicted greater helping behavior [β = 0.30, *t*(45) = 2.14, *p* = 0.04], whereas the path between the self-affirmation manipulation predicting helping behavior was no longer significant [β = 0.21, *t*(45) = 1.52, *p* = 0.14; **Figure 2**]. The significance of the indirect (mediating) effects of self-compassion was confirmed using an SPSS bootstrapping procedure (Preacher and Hayes, 2004); the indirect effect estimate of feelings of self-compassion was 0.43, with the 95% confidence interval not encompassing 0 (0.06–1.01), indicating a significant mediation effect. We also tested whether feelings of compassion mediate the relationship between self-affirmation condition and charitable giving on the spending survey. Controlling for family income, greater feelings of self-compassion did not predict increased hypothetical charitable giving [β = -0.10, *t*(48) = -0.78, *p* = 0.44], and the path between the self-affirmation manipulation predicting charitable giving remained significant [β = 0.35, *t*(48) = 2.54, *p* = 0.01].

### TESTING ALTERNATIVE SOCIAL CONNECTION ACCOUNTS OF SELF-AFFIRMATION EFFECTS

As suggested by previous research (Crocker et al., 2008), we tested whether changes in single item measures of “loving” and “connected” collected before and after the self-affirmation writing (**Figure 1**) could explain how self-affirmation increased helping behavior to the self-collapse incident. Our findings did not support a loving feelings or a feelings of connection mechanism for pro-social behavior; there were no significant self-affirmation condition differences on change in the single items “loving” [one-way ANOVA: *F*(1,50) = 1.72, *p* = 0.20] or “connected” [one-way ANOVA: *F*(1,50) = 0.34, *p* = 0.56], or the combination of “loving” and “connected” [one-way ANOVA: *F*(1,50) = 1.28, *p* = 0.26; pre-writing α = 0.74 and post-writing α = 0.75]. Specifically, there were no differences in post-pre-writing change in feeling “loving” between the self-affirmation (*M* = -0.08*,* SD = 0.81) and control (*M* = 0.22, SD = 0.85) groups, or feeling “connected” (self-affirmation: *M* = -0.04, SD = 0.89; control: *M* = 0.11, SD = 0.97; **Figure 1**).

Alternatively, as suggested by previous theorizing and research (Sherman and Cohen, 2006), we tested whether changes in overall state positive affect could explain increased helping behavior (Isen and Levin, 1972). We created a composite measure of state positive affect (five items: hopeful, secure, joyful, confident, and open; pre α = 0.77, post α = 0.84) before and after the affirmation writing manipulation. The self-affirmation group did not have greater increases in general positive affect [as assessed by a one-way ANOVA on the composite state positive affect change score: *F*(1,50) = 0.05, *p* = 0.83] compared to the control group, indicating that changes in state positive affect is not a viable mediator.

## STUDY 1 DISCUSSION

In Study 1 we found that self-affirmation increased feelings related to state self-compassion, and these feelings statistically explained how self-affirmation increased pro-social behavior to a shelf-collapse event. Self-affirmation also increased desire for charitable giving, but we were not able to shed light on the process explaining this effect in Study 1. And notably, although Study 1 was appropriately powered to test main effects of self-affirmation on self-compassion and helping outcomes, it was underpowered to test potential mediating pathways. Nonetheless, Study 1 provided the first test of sensitive pre-post-affirmation changes in affective mechanisms (including self-compassion) of behavioral helping to a shelf-collapse incident (see **Figure 1**). Our results provide preliminary evidence that self-affirmation increases compassionate feelings compared to the control writing exercise. In accordance with the self-compassion perspective, affirmation increased compassionate feelings (e.g., sympathy) but also decreased self-criticism dimensions (e.g., critical; consistent with theoretical accounts of self-compassion, Neff, 2003a). Though our results do not suggest that feelings of love or connection or general positive affect mediate the effects of self-affirmation on pro-social behavior, we can not definitively rule out that possibility.

One limitation of Study 1 is that we did not use a validated measure of state self-compassion, but constructed a composite measure of compassionate feelings using theoretically consistent items available from the administered affect checklist. It is possible that our composite self-compassion measure in Study 1 indexes other constructs besides self-compassion. To address this limitation, we conducted Study 2 to test whether self-affirmation increases self-compassion using a validated behavioral measure of self-compassionate feelings in response to a storytelling task (Leary et al., 2007). Moreover, a limitation of our Study 1 findings, like previous studies (Crocker et al., 2008), is that an alternative compassion explanation can be offered – specifically, it is unclear whether the increase in compassionate feelings in Study 1 are directed toward the self (consistent with our self-compassion account) or toward others (or toward both the self and others). Study 2 directly tests this self vs. other-focused compassion account of self-affirmation by manipulating whether participants provide ratings of their compassionate feelings toward their own storytelling video (self) or to the video of a peer (other). Study 2 also provided an opportunity to extend our self-compassion account by testing the role of trait self-compassion in moderating self-affirmation effects (we did not include a trait self-compassion individual difference measure in Study 1, a study limitation). Specifically, participants in Study 2 completed a measure of trait self-compassion (Neff, 2003b; Raes et al., 2011) to test whether self-affirmation effects on self-compassion are moderated by trait self-compassion, such that self-affirmation increases self-compassionate feelings most in participants who have pre-existing low levels of trait self-compassion.

## STUDY 2 METHOD

### PARTICIPANTS

This research was approved by the Carnegie Mellon University Institutional Review Board. Seventy-seven Carnegie Mellon students and community members (*N* = 77) were recruited (52% female; age: *M* = 21.0, SD = 2.2; 56% Caucasian, 23% Asian, 8% African American, 3% Mixed, 10% Other) in exchange for psychology class credit or $8. Participants were randomly assigned to the self-affirmation condition (*N* = 39) or control condition (*N* = 38), and to the self-video (*N* = 37) or other-video (*N* = 40) condition in a 2 × 2 between-subjects factorial design. A G^*^Power analysis indicates that at 80% power, 73 subjects are needed to detect a large overall effect with this 2 × 2 design and a continuous moderator variable (trait self-compassion).

### PROCEDURE AND DESIGN

Experimenters remained blind to participants’ affirmation condition during the experimental session, following procedures as in Study 1. Following procedures from recent self-compassion research (Leary et al., 2007, Study 4), participants arrived at the lab one-at-a-time for a study they believed explored the influence of adults’ moods on story telling. After providing written informed consent, participants completed individual difference baseline measures, including trait self-compassion (Neff, 2003b; Raes et al., 2011). Specifically, participants completed the 12-item Self-Compassion Scale – Short Form, which measures the frequency of self-compassionate feelings on a day to day basis (anchored 1 = *almost never* to 5 = *almost always*). Items were averaged to form a composite measure of trait self-compassion, with negative items reverse-scored (α = 0.86; Neff, 2003b; Raes et al., 2011). Trait self-compassion was embedded among two other exploratory baseline questionnaires: the NEO Five-Factor Inventory (NEO-FFI) Extraversion subscale (Costa and McCrae, 1992), and the Dispositional Positive Emotions Scale (DPES) Compassion subscale (Shiota et al., 2006). Then, following existing procedures for testing compassionate feelings (Leary et al., 2007, Study 4), participants were videotaped while telling an extemporaneous children’s story beginning with, “Once upon a time, there was a little bear…” for 90 s. Participants, who believed we were collecting pilot data for an unrelated study, next completed a 3-min self-affirmation or control writing exercise as described in Study 1. Additionally, participants completed a 4-item manipulation check (α = 0.97) assessing whether the writing exercise was important to their self-identity. Specifically, participants rated the personal importance of the value they wrote about on a 6-point Likert scale (*strongly disagree – strongly agree*; i.e., “*This value is an important part of who I am*;” “*In general, I try to live up to this value*”).

Then, participants were randomly assigned to either watch their own video or a female study confederate’s video, whom participants believed to be the previous study participant. After watching the storytelling video, participants completed an 8-item measure of how they felt while watching the (self or peer) video, which served as our primary measure of compassionate feelings. Specifically, participants rated eight feeling adjectives [*relaxed, happy, sad (reverse-scored), proud, embarrassed (reverse-scored), irritable (reverse-scored), nervous (reverse-scored), peaceful*] using 7-point Likert scales (1 = *not at all* to 7 = *extremely*). These items were summed to create a composite measure of compassionate feelings toward the self or other video (α = 0.83), with higher scores referencing higher compassionate feelings. Importantly, by asking participants, “*How did you feel while watching your [the] video*?” we were able to specifically probe feelings of self-compassion (or other-directed compassion) in response to this mildly embarrassing, impromptu storytelling playback. More positive feelings result from feelings of compassion, and less positive feelings reflect negative judgments and a critical response to the video. To evaluate the specificity of the self-compassionate feelings account, participants also completed a 9-item measure of their social perceptions in response to watching the storytelling video (see Leary et al., 2007, Study 4). Participants were asked to rate how they (or the peer) appeared in the video on nine performance dimensions [*awkward (reverse-scored), confident, nervous (reverse-scored), creative, reasonable, competent*, *attractive, foolish (reverse-scored)*, *likable*] using 7-point Likert scales (1 = *not at all* to 7 = *extremely*) in response to the question, “*How do you think you [the other participant] appeared on the video*?” Like the compassionate feelings composite measure, the nine items were summed to create a composite measure of performance perceptions toward the self or other video (α = 0.83), with higher scores referencing higher social perceptions of performance during the storytelling task. Thus, we were able to measure two distinct aspects of self-compassion (and other-directed compassion): feelings of (self-) compassion and performance perceptions in response to the storytelling video. These behavioral ratings of self-compassion are positively related to trait self-compassion in previous work (Leary et al., 2007, Study 4). Participants completed a final demographics measure before being probed for suspicion, fully debriefed, and dismissed.

## STUDY 2 RESULTS

A manipulation check confirmed that participants in the experimental condition identified with their chosen value and found meaning through the writing exercise as compared to the control group. Affirmed participants strongly agreed that the value they wrote about was important to their self-identity (*M* = 5.67; SD = 0.39), while control participants disagreed (*M* = 3.40, SD = 0.93) [*F*(1,73) = 196.32, *p* < 0.0005], indicating that affirmed participants found personal value in their topic.

**FIGURE 3:**
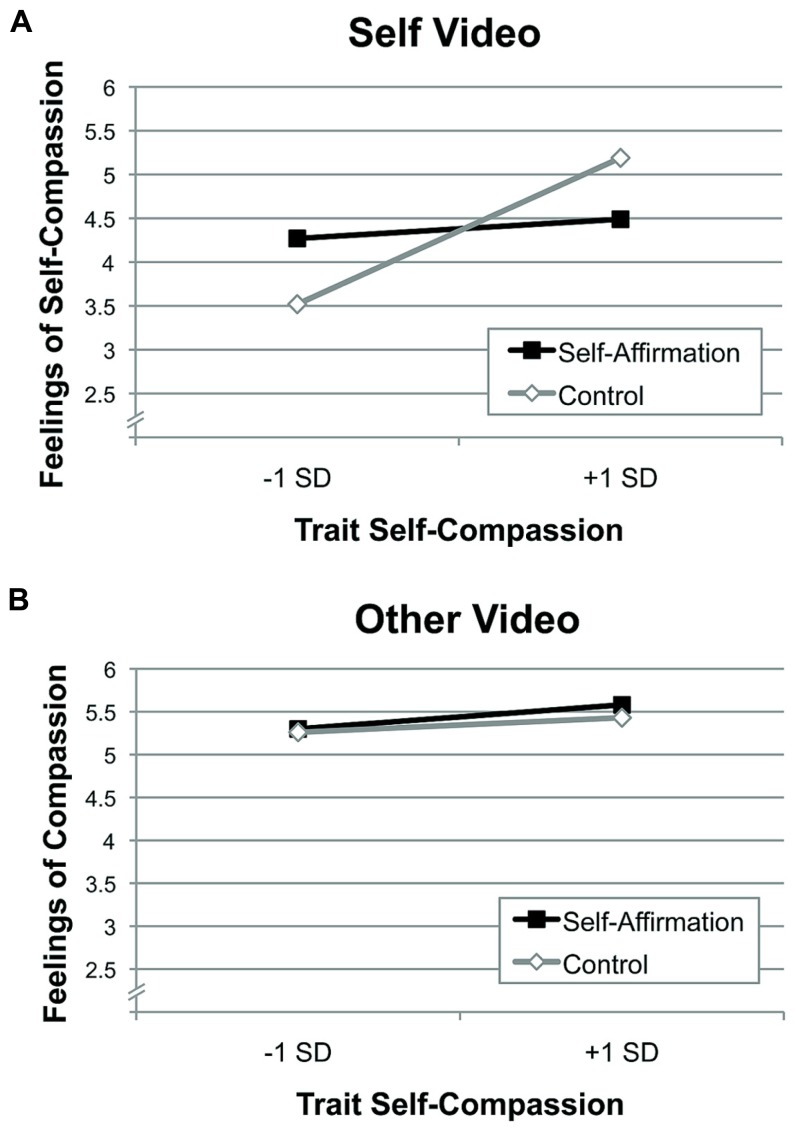
**Study 2 (A) self- vs. **(B)** other-directed feelings of compassion, by self-affirmation condition (self-affirmation vs. control), and by trait self-compassion**.

We tested two predictions in Study 2: (1) whether self-affirmation increased feelings of self-compassion but not compassionate feelings toward others, and (2) whether trait self-compassion moderates the self-affirmation self-compassion effect, such that self-affirmation would be more likely to increase self-compassionate feelings among participants who had pre-existing low levels of trait self-compassion. To test these predictions, we conducted a multiple regression analysis that modeled the self-affirmation × video condition interaction, and the 3-way trait self-compassion × self-affirmation × video condition interaction. Specifically, this multiple regression analysis included the trait self-compassion continuous predictor variable, self-affirmation condition (self-affirmation = 1 or control = 0), and video condition (self = 1 or other video = 0) as predictor variables, along with their two-way interactions, and one 3-way interaction term. **Table 1** provides the results of this multiple regression analysis for compassionate feelings to the storytelling video, and **Figure 3** visually depicts the results. Notably, this regression analysis revealed a significant main effect of video condition, such that those who watched their own video had lower feelings of compassion than those who watched the confederate’s video [β = -2.31, *t*(69) = -3.96, *p* < 0.005]. Moreover, we observed a significant trait self-compassion × video condition interaction, showing that participants lower in trait self-compassion rated their own video less favorably relative to participants higher in trait self-compassion (whereas trait self-compassion did not impact ratings of a peer’s video). This result conceptually replicates previous research showing that trait self-compassion moderates behavioral self-compassion to a storytelling video (Leary et al., 2007).

**Table 1:** Multiple regression analysis results for feelings of self-compassion in Study 2.

	Beta	*t*-statistic	*p*-value
Affirmation condition	-0.84	-0.14	0.89
Video condition	-2.31	-3.96	**<0.005**
Trait self-compassion	0.09	0.53	0.60
Affirmation condition × video condition	1.63	2.20	**0.03**
Affirmation condition × self-compassion	0.14	0.24	0.81
Video condition × self-compassion	1.89	3.21	**0.002**
Affirmation condition × video condition × self-compassion	-1.74	-2.33	**0.02**

*Significant p-values (*p* < 0.05) are in bold.
*

The multiple regression analysis supports both of the primary Study 2 self-compassion predictions. First, we observed a significant self-affirmation × video condition interaction [β = 1.63, *t*(69) = 2.20, *p* = 0.03], such that participants who completed a self-affirmation activity had more feelings of compassion toward the self video compared to the control writing group participants, whereas self-affirmation did not influence other-directed feelings of compassion in rating a peer storytelling video. As a follow-up test of self-affirmation effects on self-compassion in general, we ran a t-test of the subsample of participants (*N* = 37) who viewed their own storytelling video. Mean feelings of self-compassion were higher after self-affirmation (*M* = 4.38, SE = 0.22) than control writing (*M* = 4.26, SE = 0.25), though this analysis was not statistically significant [*t*(35) = -0.36, *p* = 0.73]. This 2-way self-affirmation × video condition interaction result was qualified by the predicted 3-way self-affirmation × video condition × trait self-compassion interaction [β = -1.74, *t*(69) = -2.33, *p* = 0.02]. Specifically, self-affirmation increased feelings of self-compassion (but not other-directed feelings of compassion toward a peer video) in participants with lower pre-existing trait levels of self-compassion (**Figure 3**). This result is consistent with the prediction that self-affirmation can help boost deficient self-resources, in this case increasing feelings of self-compassion in participants with lower trait self-compassion.

We conducted an identical multiple regression analysis with the social performance perceptions measure as the dependent variable. Specifically, this multiple regression analysis tests whether the previous findings related to feelings of compassion also extend to affect social perceptions of performance on the storytelling task (e.g., “how competent did you [the other participant] appear in the video?”). The regression results are depicted in **Table 2**. Like the compassion feelings measure, participants rated the performance of the peer video higher than the self-video [a main effect of video condition: β = -1.83, *t*(69) = -2.89, *p* = 0.005], and trait self-compassion moderated performance perceptions of the videos [trait self-compassion × video condition interaction: β = 1.50, *t*(69) = 2.35, *p* = 0.02], such that participants lower in trait self-compassion had lower performance perceptions of their own video (but trait self-compassion did not impact peer video ratings). As shown in **Table 2**, our results indicate some specificity of the self-affirmation effects to self-compassionate feelings (and not to more general social perceptions): self-affirmation did not significantly impact social performance perceptions (there was no significant self-affirmation × video condition interaction, and no 3-way interaction; **Table 2**), though this study may have been underpowered to detect subtle influences of self-affirmation on social performance perceptions.

**Table 2:** Multiple regression analysis results for social performance perceptions in Study 2. (*p* < 0.05)

	Beta	*t*-statistic	*p*-value
Affirmation condition	-0.53	-0.81	0.42
Video condition	-1.83	-2.89	<**0.005**
Trait self-compassion	0.07	0.37	0.72
Affirmation condition × video condition	1.08	1.33	0.19
Affirmation condition × self-compassion	0.62	0.94	0.35
Video condition × self-compassion	1.50	2.35	**0.02**
Affirmation condition × video condition × self-compassion	-1.26	-1.55	0.13

*Significant p-values (*p* < 0.05) are in bold.*

## STUDY 2 DISCUSSION

Study 2 provides a first indication that self-affirmation increases feelings of self-compassion using an established storytelling task-based measure. This result was specific to self-compassion; self-affirmation did not affect other-directed feelings of compassion toward a peer video. Moreover, the effect of self-affirmation on feelings of self-compassion was moderated by trait self-compassion, such that self-affirmation boosted feelings of self-compassion toward the storytelling video in those who were low in trait self-compassion. These findings help clarify the Study 1 findings where it was unclear whether the compassionate feelings encouraging helping behavior were directed at the self or directed out toward others. Here we find evidence that self-affirmation fosters compassionate feelings for the self but not toward a peer, which is consistent with the self-compassion account. However, the use of a single confederate video may not have been optimally matched to real participants’ self videos, perhaps differing on unmeasured variables despite our best efforts to film this peer video under matched conditions (the female research assistant in the video had no chance to practice or provide multiple takes, and was similarly embarrassed during the task as the study participants).

Study 2 also provides some specificity around the relationship between self-affirmation and self-compassionate feelings; we did not find evidence that self-affirmation affected more general performance perceptions of the self or peer storytelling videos, though our study may be underpowered to detect subtle differences in this dimension of self-compassion. Though we do not definitively rule out this possibility, our results suggest that self-affirmation effects may be specific to affective measures of self-compassion, which is consistent with the affective change in self-compassion we observed in Study 1.

## GENERAL DISCUSSION

The present findings provide an initial indication for a self-compassion account of self-affirmation effects. Specifically, we find in two studies that self-affirmation can increase self-compassionate feelings, and that these feelings foster more pro-social behaviors (in Study 1). Moreover, Study 2 provides direct evidence that these compassionate feelings are directed toward the self (and not toward others) and are specific to affective perceptions (and not general performance perceptions). Study 2 also highlights an important moderating role of trait self-compassion, suggesting that self-affirmation enhances feelings of self-compassion specifically for those dispositionally deficient in this resource. However, while we believe that self-compassion is a promising mechanism for self-affirmation effects, more research is needed to test these conclusions.

We believe that the most important contribution this work makes is in relating the two leading theoretical perspectives on the mechanisms of self-affirmation, offering one account for how the *self-resources* and *self-transcendence* perspectives may be linked, via self-compassion. Overall, our results suggest a process through which self-affirmation may mobilize self-resources (specifically, self-compassion), which in turn allow for self-transcendence (and pro-social focus on others).

Like the self-resources account, our findings indicate that self-affirmation boosts one’s self-image by increasing positive self-feelings, but provide additional specificity about the nature of these feelings; self-affirmation increases feelings related to self-compassion (e.g., sympathy, trust, and less criticism; Study 1). Like self-esteem, self-compassion predicts positive feeling states, but is distinguished by its more stable relationship to self-worth, independent of positive or negative outcomes (Neff and Vonk, 2009). Consistent with this, in response to a potentially embarrassing video of oneself, affirmed participants maintained positive self-feelings (Study 2). The effect of self-affirmation writing on self-compassion may explain why few studies have shown that self-affirmation increases general feelings of state self-esteem or positive affectivity (Sherman and Cohen, 2006).

Like the self-transcendence account, our Study 1 outcome showing that self-affirmation increases pro-social behavior is consistent with the idea that self-affirmation fosters social connectedness (Crocker et al., 2008; Burson et al., 2012), but our Study 2 findings suggest that these compassionate feelings may be directed toward the self (and not toward a peer). However, further research is necessary to clarify this finding. In Study 1, feelings of compassion boost pro-social behavior, but in Study 2, other-directed feelings of compassion are not impacted by self-affirmation writing. A ceiling effect may explain this seeming difference; the confederate “other” storytelling video we used was rather high quality, and may not have solicited a need for compassion, thus explaining the lack of variability in participants’ responses across conditions. Or, it’s possible that watching a peer’s slightly embarrassing video might not elicit a compassionate vs. judgmental response comparable to feelings of self-compassion vs. self-judgment in response to the self video. Future work is needed to establish whether self-affirmation also increases compassionate feelings for others in need, perhaps using different methods to compare self- vs. other-directed compassionate responses.

Our research provides a promising indication of the pro-social benefits of self-affirmation and self-compassionate feelings. It is perhaps not surprising that feelings of compassion have been associated with increased helping behavior (Mikulincer et al., 2005; Hutcherson et al., 2008; Piff et al., 2010, Study 4), but no published studies (to our knowledge) have tested whether self-compassionate feelings can mobilize helping behavior. His Holiness The Dalai Lama poignantly stated this possibility when he said “*If you don’t love yourself, you cannot love others. You will not be able to love others. If you have no compassion for yourself then you are not able of developing compassion for others.”* Our study provides an initial experimental demonstration of this idea; we find that increasing feelings of self-compassion (via a self-affirmation activity) can mobilize helping behaviors (to a shelf-collapse incident). Thus self-affirmation may address internally derived self-threats (increasing self-compassion), which in turn allow one to transcend these self-concerns and focus on helping others. Our work joins previous work showing that self-compassion may also act as a buffer to self-threatening events and negative emotions (Neff, 2003a; Leary et al., 2007).

## CONCLUSION

Our present findings suggest that self-affirmation may increase feelings of self-compassion, and that self-compassion may be a promising new mechanism for a potentially broad range of self-affirmation effects. More research is needed, but the present research provides an initial suggestion that affirming an important personal value increases feelings of self-compassion for mobilizing a pro-social self.

## AUTHOR CONTRIBUTIONS

Emily K. Lindsay contributed to study conception and design; data acquisition, analysis, and interpretation; and manuscript drafts and revisions. J. David Creswell contributed to study conception and design; data interpretation; and manuscript drafts and revisions.

## Conflict of Interest Statement

The authors declare that the research was conducted in the absence of any commercial or financial relationships that could be construed as a potential conflict of interest.

## References

[B1] Baron R. M. Kenny D. A. (1986). The moderator–mediator variable distinction in social psychological research: conceptual, strategic, and statistical considerations. *J. Pers. Soc. Psychol.* 51 1173–1182 10.1037/0022-3514.51.6.1173 3806354

[B2] Burson A. Crocker J. Mischkowski D. (2012). Two types of value-affirmation implications for self-control following social exclusion. *Soc. Psychol. Personal. Sci.* 3 510–516 10.1177/1948550611427773

[B3] Cohen G. L. Garcia J. Apfel N. Master A. (2006). Reducing the racial achievement gap: a social-psychological intervention. *Science* 313 1307–1310 10.1126/science.1128317 16946074

[B4] Cohen G. L. Sherman D. K. (2014). The psychology of change: self-affirmation and social psychological intervention. *Annu. Rev. Psychol.* 65 333–371 10.1146/annurev-psych-010213-115137 24405362

[B5] Costa P. T. McCrae R. R. (1992). *Revised NEO Personality Inventory (NEO PI-R) and NEO Five-Factor Inventory (NEO-FFI)* Vol. 101. Odessa: Psychological Assessment Resources

[B6] Creswell J. D. Dutcher J. M. Klein W. M. P. Harris P. R. Levine J. M. (2013). Self-affirmation improves problem-solving under stress. *PLoS ONE* 8:e62593 10.1371/journal.pone.0062593 PMC3641050 23658751

[B7] Creswell J. D. Welch W. T. Taylor S. E. Sherman D. K. Gruenewald T. L. Mann T. (2005). Affirmation of personal values buffers neuroendocrine and psychological stress responses. *Psychol. Sci.* 16 846–851 10.1111/j.1467-9280.2005.01624.x 16262767

[B8] Crocker J. Niiya Y. Mischkowski D. (2008). Why does writing about important values reduce defensiveness? *Psychol. Sci.* 19 740–747 10.1111/j.1467-9280.2008.02150.x 18727791

[B9] Hutcherson C. A. Seppala E. M. Gross J. J. (2008). Loving-kindness meditation increases social connectedness. *Emotion* 8 720–724 10.1037/a0013237 18837623

[B10] Isen A. M. Levin P. F. (1972). Effect of feeling good on helping: cookies and kindness. *J. Pers. Soc. Psychol.* 21 384–388 10.1037/h0032317 5060754

[B11] Koole S. L. Smeets K. Van Knippenberg A. Dijksterhuis A. (1999). The cessation of rumination through self-affirmation. *J. Pers. Soc. Psychol.* 77 111–125 10.1037/0022-3514.77.1.111

[B12] Leary M. R. Tate E. B. Adams C. E. Batts Allen A. Hancock J. (2007). Self-compassion and reactions to unpleasant self-relevant events: the implications of treating oneself kindly. *J. Pers. Soc. Psychol.* 92 887–904 10.1037/0022-3514.92.5.887 17484611

[B13] McQueen A. Klein W. M. (2006). Experimental manipulations of self-affirmation: a systematic review. *Self Identity* 5 289–354 10.1080/15298860600805325

[B14] Mikulincer M. Shaver P. R. Gillath O. Nitzberg R. A. (2005). Attachment, caregiving, and altruism: boosting attachment security increases compassion and helping. *J. Pers. Soc. Psychol.* 89 817 10.1037/0022-3514.89.5.817 16351370

[B15] Miyake A. Kost-Smith L. E. Finkelstein N. D. Pollock S. J. Cohen G. L. Ito T. A. (2010). Reducing the gender achievement gap in college science: a classroom study of values affirmation. *Science* 330 1234–1237 10.1126/science.1195996 21109670

[B16] Neff K. (2003a). Self-compassion: an alternative conceptualization of a healthy attitude toward oneself. *Self Identity* 2 85–101 10.1080/15298860309032

[B17] Neff K. (2003b). The development and validation of a scale to measure self-compassion. *Self Identity* 2 223–250 10.1080/15298860309027

[B18] Neff K. D. Vonk R. (2009). Self-compassion versus global self-esteem: two different ways of relating to oneself. *J. Pers.* 77 23–50 10.1111/j.1467-6494.2008.00537.x 19076996

[B19] Piff P. K. Kraus M. W. Côté S. Cheng B. H. Keltner D. (2010). Having less, giving more: the influence of social class on prosocial behavior. *J. Pers. Soc. Psychol.* 99 771–784 10.1037/a0020092 20649364

[B20] Preacher K. J. Hayes A. F. (2004). SPSS and SAS procedures for estimating indirect effects in simple mediation models. *Behav. Res. Methods Instrum. Comput.* 36 717–731 10.3758/BF03206553 15641418

[B21] Raes F. Pommier E. Neff K. D Van Gucht D. (2011). Construction and factorial validation of a short form of the Self-Compassion Scale. *Clin. Psychol. Psychother.* 18 250–255 10.1002/cpp.702 21584907

[B22] Schmeichel B. J. Vohs K. (2009). Self-affirmation and self-control: affirming core values counteracts ego depletion. *J. Pers. Soc. Psychol.* 96 770–782 10.1037/a0014635 19309201

[B23] Schnall S. Roper J. (2012). Elevation puts moral values into action. *Soc. Psychol. Personal. Sci.* 3 373–378 10.1177/1948550611423595

[B24] Sherman D. K. Cohen G. L. (2006). “The psychology of self-defense: Self-affirmation theory,” in Vol. 38 *Advances in Experimental Social Psychology* ed. Zanna M. P. (San Diego, CA: Academic Press) 183–242 10.1016/S0065-2601(06)38004-5

[B25] Sherman D. K. Hartson K. A. (2011). “Reconciling self-protection with self-improvement,” in *Self-Affirmation Theory* eds Alicke M. Sedikides C. (New York, NY: Guilford Press).

[B26] Shiota M. N. Keltner D. John O. P. (2006). Positive emotion dispositions differentially associated with Big Five personality and attachment style. *J. Posit. Psychol.* 1 61–71 10.1080/17439760500510833

[B27] Thomaes S. Bushman B. J. de Castro B. O. Reijntjes A. (2012). Arousing “gentle passions” in young adolescents: sustained experimental effects of value affirmations on prosocial feelings and behaviors. *Dev. Psychol.* 48 103–110 10.1037/a0025677 21967565

[B28] Watson D. Clark L. A. Tellegen A. (1988). Development and validation of brief measures of positive and negative affect: the PANAS scales. *J. Pers. Soc. Psychol.* 54 1063–1070 10.1037/0022-3514.54.6.1063 3397865

